# Area summation in human vision at and above detection threshold

**DOI:** 10.1098/rspb.2007.0957

**Published:** 2007-09-13

**Authors:** Tim S Meese, Robert J Summers

**Affiliations:** School of Life and Health Sciences, Aston University Birmingham B47ET, UK

**Keywords:** contrast gain control, spatial summation, dipper function, suppression, psychophysics

## Abstract

The initial image-processing stages of visual cortex are well suited to a local (patchwise) analysis of the viewed scene. But the world's structures extend over space as textures and surfaces, suggesting the need for spatial integration. Most models of contrast vision fall shy of this process because (i) the weak area summation at detection threshold is attributed to probability summation (PS) and (ii) there is little or no advantage of area well above threshold. Both of these views are challenged here. First, it is shown that results at threshold are consistent with linear summation of contrast following retinal inhomogeneity, spatial filtering, nonlinear contrast transduction and multiple sources of additive Gaussian noise. We suggest that the suprathreshold loss of the area advantage in previous studies is due to a concomitant increase in suppression from the pedestal. To overcome this confound, a novel stimulus class is designed where: (i) the observer operates on a constant retinal area, (ii) the target area is controlled within this summation field, and (iii) the pedestal is fixed in size. Using this arrangement, substantial summation is found along the entire masking function, including the region of facilitation. Our analysis shows that PS and uncertainty cannot account for the results, and that suprathreshold summation of contrast extends over at least seven target cycles of grating.

## 1. Introduction

A fundamental requirement of the primate visual system is the building of higher-order representations of spatially extensive textures, surfaces and objects from the initial feature/filter code in primary visual cortex. One step towards this goal is to extend the principle of neuronal convergence found in the retina and lateral geniculate nucleus to visual cortex (Olzak & Thomas 1999). In this way, area summation of luminance contrast can be achieved by summing the outputs of multiple local filters (e.g. striate cells). In fact, a substantial body of work has found that detection thresholds decrease with the area of a sine-wave grating, providing good evidence for an area summation process of some kind (e.g. Howell & Hess 1978; Robson & Graham 1981; Rovamo *et al*. 1993; Meese 2004; Foley *et al*. 2007).

### (a) Signal combination or probability summation?

But does area summation involve the signal combination process described above? A computationally distinct alternative is ‘probability summation’ (PS), where the greater the number of detectors stimulated, the greater the probability that the stimulus will be detected. The PS nomenclature pertains to earlier psychophysical work built around a high threshold model of the detection process (e.g. Sachs *et al*. 1971; Robson & Graham 1981). This model assumes a formal relation between per cent correct (the psychometric function) and the probability that the stimulus strength exceeded detection threshold (an output nonlinearity). From this it follows that the benefit from PS between independent detectors depends on the slope of the psychometric function (Quick 1974).

More generally, a convenient expression for summation in a variety of situations is Minkowski summation: ${\text{resp}}_{\text{overall}}=\sum_{i=1\;:\;n}{\left({|\text{resp}}_{i}|m\right)}^{1/m}$, where resp_*i*_ is the contrast response of the *i*th mechanism and *m* (sometimes called the Minkowski exponent) controls the level of summation (which decreases as *m* increases). From Quick (1974) it follows that if the psychometric function is a Weibull function, then its slope parameter (*β*) equals the Minkowski exponent (*m*) when the relation between resp_*i*_ and stimulus strength (e.g. contrast) is linear and summation is PS. When resp_*i*_ is constant across *i*, a property of Minkowski summation is that: *m*′=−1/*m*, where *m*′ is the log–log threshold slope against the number of detecting mechanisms, *n*. Empirical estimates of the psychometric slope are typically $3\leq \hat{\beta }\leq 4$ at detection threshold (e.g. Mayer & Tyler 1986), and area summation is quite gentle beyond a few cycles of grating: *m*′∼−1/3 to −1/4 (Robson & Graham 1981). This close empirical relation between Minkowski exponent (*m*) and psychometric slope ($\hat{\beta }$; Watson 1979; Meese & Williams 2000) has been taken as evidence that area summation of contrast arises through PS (Robson & Graham 1981).

However, as high threshold theory is discredited (e.g. Nachmias 1981), the theoretical basis for a Minkowski implementation of PS is undermined. A contemporary signal detection framework for PS was developed by Tyler & Chen (2000). They built their analysis around the two-interval forced-choice (2IFC) design of psychophysical experiments and assumed a linear contrast transducer for simplicity. The observer is assumed to select the interval containing the mechanism with the largest (MAX) response. This analysis found that *m*′∼−1/4 in several situations (sometimes called a fourth-root rule), justifying the use of *m*=4 in Minkowski summation, but in general *m*≠*β* in this framework.

In spite of the empirical success of the fourth-root rule and its association with PS, a signal combination framework remains viable. We develop this in §3*a* and appendix A.

### (b) Summation above threshold?

Although contrast sensitivity improves with grating area around detection threshold, empirical summation is diminished or abolished above threshold (Legge & Foley 1980; Näsänen *et al*. 1998; McIlhagga & Pääkkönen 1999; Meese 2004; Chirimuuta & Tolhurst 2005; Meese *et al*. 2005), suggesting that the integration process is made inoperative (e.g. Legge & Foley 1980; Swanson *et al*. 1984). This also fuelled support for the idea that summation at threshold is due to PS (rather than signal combination), because it was much easier to see a method by which this type of summation could be disabled; if noise were to become correlated above threshold, there would be no benefit in having multiple detecting mechanisms (Legge & Foley 1980). However, another possibility is that the benefits of the area summation process are offset by an equal and opposite effect of suppression that increases with the size of the pedestal (Bonneh & Sagi 1999; Meese *et al*. 2005). With this in mind, Meese (2004) attempted to isolate the summing process by investigating different combinations of small (S) and large (L) target and pedestal diameters. For two out of three observers, the masking functions for all three target/pedestal configurations (SS, SL and LL) converged at high pedestal contrasts, confirming that area summation does not operate unhindered well above threshold. The modelling accommodated this by a process of suprathreshold suppression, but the failure to produce a compelling empirical illustration of suprathreshold summation leaves the status of the summation process unresolved.

### (c) Aims

Our main aim here was to devise novel stimulus conditions that would reveal the putative excitatory summation process empirically. The SL and LL conditions of Meese (2004) were an improvement on earlier comparisons between SS and LL because the pedestal size was not confounded with target size. However, the spatial extent of excitatory integration probably differed across the two conditions (Meese 2004). To avoid this complication, it is preferable that the target size is varied within a fixed region of putative integration in an attempt to tap a common mechanism or process. We take up this challenge here by designing a stimulus class that appears to meet this requirement.

A second issue is that the analysis in Meese (2004) did not address whether the underlying process was one of PS or signal combination. We develop a model of signal combination in experiments 1 and 2, and reject the PS model in experiment 2 by extending the analysis to include the slope of the psychometric function (Tyler & Chen 2000).

## 2. Methods

### (a) Equipment

Stimuli were displayed from the framestores of Cambridge Research Systems (CRS) stimulus generators operating in pseudo 14- or 15-bit mode and controlled by a P. C. The monitors were a Sony Multiscan 20SEII for experiment 1, and either an Eizo Flex scan F553-M or a Clinton Monoray (observers Y. R. and A. S. P.) for experiments 2 and 3. Mean luminance was 61 cd m^−2^ for the Sony and 50 cd m^−2^ for the Eizo and Clinton (The Clinton was viewed through CRS ferro-electric (FE-1) shutter goggles which remained open on all frames for both eyes). All three monitors had a frame rate of 120 Hz. In experiments 2 and 3 the image refresh rate was 60 Hz. Look up tables were used to perform gamma correction to ensure linearity over the full range of stimulus contrasts. Observers sat at a viewing distance of 72.5 cm in experiment 1 and 51.5 cm in experiments 2 and 3, with their head in a chin and headrest, and viewed the stimuli binocularly.

### (b) Stimuli

The three different types of stimuli used in experiments 2 and 3 are shown in figure 1. The full stimulus (figure 1*a*) was a horizontal sine-wave grating in sine-phase with the centre of the display, and had a spatial frequency of 2.5 cycles per degree. It was modulated by a circular raised cosine function with a central plateau of 8° and a blurred boundary of 1°, giving a full-width at half-height of 9°. The check stimuli (figure 1*b*,*c*) were identical to the full stimulus, except that they were modulated by a ‘raised-plaid’ envelope. The plaid was the sum of two sine-wave grating components with orientations of ±45° and a spatial frequency of 0.5 cycles per degree, each with contrasts of 0.5. This gave minima and maxima of −1 and 1, respectively. The envelope was then ‘raised’ by adding 1 to each point and dividing by 2 to give minima and maxima of 0 and 1. With this arrangement, there are 7.07 cycles of carrier grating for every two checks (i.e. one cycle of a vertical cross-section through the envelope).

In figure 1*b* the modulator is in cosine-phase with the centre of the display and in figure 1*c* it is in -cosine phase. These stimuli are given the nominal titles of ‘white’ and ‘black’ checks, respectively (a reference to the magnitude of the modulator at the centre of the display). Note that the physical sum of the stimuli in figure 1*b*,*c* is equal to the full stimulus in figure 1*a*.

In experiment 1 the stimuli were full stimuli and ‘white’ check stimuli. However, they differed from those in figure 1 in three ways: the carrier grating was oriented vertically; the blurring of the edges extended over only 2 pixels (4.8 arcmin); and their diameter varied across conditions. There were eight different sizes of the full stimulus and four different sizes of the check stimulus. The smallest stimulus had a full diameter of 14 pixels (10 for the central plateau of one cycle, plus two on each side for the blurred boundary). The full set of stimuli is provided in electronic supplementary material 3.

In experiments 2 and 3 a dark square fixation point (4.8 arcmin wide) was displayed in the centre of the display throughout the experiment. In experiment 1 no fixation point was used. In all experiments, carrier contrast is expressed as Michelson contrast in percentage (i.e. *c*=100((*L*_max_−*L*_min_)/(*L*_max_−*L*_min_))) or, for consistency with previous work, in dB re 1% (=20 log_10_(*c*)).

There were four possible combinations of target on pedestal: (i) full-on-full, (ii) ‘black’ checks-on-full, (iii) ‘white’ checks-on-full, and (iv) ‘white’ checks-on-checks.

### (c) Procedure

In experiments 1 and 2, target contrast was selected by a staircase procedure. Three consecutive correct responses and a single incorrect response caused the stimulus level to be incremented and decremented by a single contrast ‘step’, respectively (Wetherill & Levitt 1965). Each condition was tested using a pair of randomly interleaved staircases. The target contrast always began well above detection threshold and each staircase terminated after 12 reversals with a step-size of 3 dB. A temporal 2IFC technique was used. In most conditions, one interval contained only the pedestal and the other the pedestal plus target. In experiment 1, the pedestal contrast was 0%. In all experiments, the onset of each 100 ms stimulus interval was indicated by an auditory tone and the duration between the two intervals was 400 ms. The observer's task was to identify the target interval using one of two buttons to indicate their response. Correctness of response was provided by auditory feedback, and the computer selected the order of the intervals randomly. For each run, data were collapsed across the two staircases and thresholds (75% correct) and standard errors were estimated by probit analysis. Each condition was run four times.

In all experiments, stimulus conditions were blocked and observers were aware of which stimulus was being used as the target. The order of conditions was random.

In experiment 3 a method of constant stimuli was used (120 trials per level). Pedestal contrasts were either 0 or 20% in different runs. A preliminary detection experiment determined sensitivity (75% correct) to full and ‘white’ check increments. In a subsequent 2IFC identification task, one interval contained a full increment and the other contained an equally detectable ‘white’ check increment. The observers' task was to select the interval containing the ‘white’ check increment.

### (d) Observers

An author (T.S.M.) was the only observer to perform experiment 1. Seven undergraduate optometry students performed experiment 2 (the main experiment) as part of their course requirement. They were D.B.D, P.C, C.M., L.M., L.W., Y.R. and A.S. P. Of these, only L.M. and L.W. performed all four conditions. Both authors (T.S.M. and R.J.S.) performed experiment 3. All observers wore their normal optical correction and had normal stereopsis.

## 3. Results and Discussion

### (a) Experiment 1: proof of concept

The filled circles in figure 2 show area summation for the full stimulus, which is a bowed function of stimulus area. The initial part of the function approximates a slope of *m*′=−1/2 on these double-log coordinates. The intermediate region is shallower and approximates a slope of *m*′=−1/4, but becomes asymptotic thereafter. This general form is similar to that found in previous studies, where area summation has been measured in the central visual field (Tootle & Berkley 1983; Garcia-Perez 1988; Rovamo *et al*. 1993; Foley *et al*. 2007).

The thick continuous and dashed curves in figure 2 are model predictions. The model is described formally in appendix A, but in brief, it operates as follows. The image is multiplied by an attenuation surface to simulate the effects of retinal inhomogeneity and convolved with sine- and cosine-phase filters matched to the spatial frequency and orientation of the carrier (see inset for sine-phase example). The response at each pixel is full-wave rectified and passed through an accelerating contrast transducer with an exponent *p*=2.4 (Legge & Foley 1980). Added to the output at each pixel is zero-mean, unit variance, Gaussian noise. This is followed by linear summation of the filtered signal and noise across the target region and to determine sensitivity. The model is deterministic and establishes the influence of multiple independent noise sources by combining their (unit) variances in the conventional way. Thus, the standard deviation of the noise∝$\sqrt{(2n})$, where *n* is the number of pixels in the target. The signal contrast is set to produce unit SNR for each target and is normalized to the detection threshold for the smallest target.

Although summation extends over the full extent of the largest stimulus in the model (50 carrier cycles), detection thresholds improve little beyond eight cycles. In the model, this is due to the effect of retinal inhomogeneity. Of more importance here is the substantial improvement (approx. 5 dB) across the two stimulus types (different symbols). In the model, this is because noise and retinal sensitivity are constant across the two stimulus types, but spatial summation of contrast results in much greater sensitivity to the full stimulus (filled circles). These model assumptions suppose that the visual system cannot switch out the less informative contributions in the low-contrast signal regions of the check stimuli where noise is dominant. The close proximity between model and data suggests that this is reasonable. These results emphasize the difference between a conventional summation experiment, where area increases with stimulus diameter (abscissa), and the new approach here, where the diameter is constant and the area is increased by filling in the low-contrast (black) patches of the stimulus (different symbols). Note that ‘filling-in’ increases the stimulus area (the sum of contrast over area) by a factor of 2, equivalent to a single tick mark along the abscissa for the conventional method (filled circles). However, the conventional method never achieves a level of summation comparable with that obtained using the filling-in method. In part, this is presumably because the conventional method confounds noise level and retinal sensitivity with area.

### (b) Experiment 2: extending the result above threshold

In experiment 2 we replicated the key result from experiment 1 (comparison across check and full stimuli) for seven other observers and extended the study above threshold. The results are shown in figure 3*a* and averaged across the two observers who performed all of the conditions (L. M. and L. W.). The filled circles are for when the full stimulus (figure 1*a*) was used as both pedestal and target and have a classic ‘dipper’ shape. The crossed squares are for when the ‘white’ checks stimulus was used as both the pedestal and the target. Although the two stimulus types have the same diameters (figure 1), the sum of contrast over area for the full stimulus is twice that of the check stimuli. Hence, we refer to the full stimulus as having a greater (signal) area than the check stimulus of corresponding size. A comparison of these two conditions replicates the classic area summation result of Legge & Foley (1980): at low pedestal contrasts there is a distinct advantage for the full stimulus, which has the greater area, but at higher pedestal contrasts the two masking functions converge. The half-filled squares are for when the target was one of the check stimuli (figure 1*b*,*c*), but the pedestal was the full stimulus. (The results were almost identical for ‘black’ and ‘white’ checks—as confirmed in figure 4*a* below—and have been averaged together.) A comparison of this with the full-on-full condition (compare circles and half-filled squares) shows the effect of fixing the pedestal area and increasing only the target area. In this case, the area advantage at detection threshold extends across the entire dipper function, providing strong evidence for a spatial summation process that remains intact across a wide range of contrasts.

Our threshold model (from experiment 1) was extended to operate across the full contrast range and provides a very good account of the general form of these three functions (figure 3*b*). It sums stimulus contrast (both pedestal and target) over area on the numerator and denominator of a contrast gain control equation,

(3.1) $${\text{resp}}_{\text{stim}}=\frac{\sum_{i=1\;:\;2n}|{\text{mech}}_{i}(\text{stim}){|}^{2.4}}{z+\sum_{i=1:2n}|{\text{mech}}_{i}(\text{stim}){|}^{2}}$$

where mech_*i*_ is the full-wave rectified contrast response of the *i*th filter element (mechanism) in the stimulus region after retinal inhomogeneity (see appendix B for details). It is well known that this general form of equation produces a dipper function (Legge & Foley 1980; Meese 2004). Furthermore, the saturation constant, *z*, ensures an area advantage at threshold because an increase in signal area impacts substantially only on the numerator. Above threshold, when the area of both target and pedestal is increased (crossed squares versus filled circles), the pedestal and target impact both the numerator and denominator and the masking functions converge (Meese *et al*. 2005). In contrast, when the pedestal area is fixed and only the target area grows (half-filled squares versus filled circles), the impact is most effective on the numerator, and area summation occurs for all pedestal contrasts.

The model (equation (3.1)) implements a blanket pooling strategy consistent with the main aim of our stimulus design (see §1*c*). The importance of this is shown in figure 3*c* where excitatory pooling has been restricted to the high-contrast regions of the checks (i.e. ‘white’ half of the image) in the checks-on-full condition (half-shaded squares). This is comparable with the restricted excitatory pooling for the SL condition in Meese (2004). In both studies this pooling strategy has the same effect: all three masking functions tend towards convergence. However, this is not consistent with the data here (figure 3*a*), suggesting that observers could not restrict excitatory integration in this way. The extra masking in figure 3*b* (compare half-shaded squares across figure 3*b*,*c*) is due to the mandatory excitatory integration over the non-target regions. We refer to this as *dilution masking*.

### (c) Summation level

Figure 4*a* shows the results averaged across a total of six observers (L.M. and L.W. from before, plus D.B. D, P.C., C.M. and A.S.P.), where the pedestal was always a full stimulus (see electronic supplementary material 2 for individual datasets). As mentioned above, the results for the ‘black’ and ‘white’ checks were almost identical (different square symbols), as were model predictions for these two conditions (not shown). Figure 4*b* shows the level of summation as a function of pedestal contrast derived from the ratios of the full condition and the average check condition in figure 4*a*. The thick dashed curve is the model prediction derived in the same way (no further free parameters). Note that for model and data, the level of summation increases slightly (by approx. 1 dB) over the first part of the function, but then asymptotes around 6 dB (a factor of 2) at higher contrasts. Thus, the level of summation is substantial across the entire contrast range for the model and these six observers.

The results for Y.R were slightly different. Although her levels of summation were similar to the others at the lower pedestal contrasts, they fell to approximately 3 dB at the higher contrasts. It is not clear why this occurred but it could be due to the use of less or more efficient pooling strategies for the full and check targets, respectively (see above). In the next experiment, the results for R.J.S. (but not T.S.M.) also show less than typical area summation above threshold.

### (d) Experiment 3: signal detection and identification

Our main proposal is that the full and check stimuli are detected by a common pooling process (e.g. equation (3.1)). Subjective reports of our observers (who were questioned during the experiment) are consistent with this view: when the checks-on-full stimulus was close to threshold, the target increment appeared to be applied to the entire pedestal. If so, it should be difficult for the observers to identify a check target in a 2IFC experiment, where equally detectable check and full increments are placed in the two intervals. An alternative hypothesis is that the different increments are detected by different mechanisms. For example, the check stimulus might be detected by a second-order mechanism sensitive to contrast modulation (Georgeson & Schofield 2002). If these involve labelled lines (Watson & Robson 1981), then observers should be able to identify the different increment types close to their thresholds (Georgeson & Schofield 2002). Figure 5 shows that this does not happen for pedestal contrasts of either 0% (figure 5*a*(i),*b*(i)) or 20% (figure 5*a*(ii),*b*(ii)). On these normalized axes, the psychometric functions for detecting the two different increment types (squares and circles) superimpose (i.e. the results for the checks condition were slid laterally). In the identification task (crosses), equally detectable full and ‘white’ check contrast increments were made in the two test intervals and observers had to identify the checks. But the contrast increment needed to do this successfully was much higher than for detection. Although this experiment does not identify the form of pooling (PS or signal combination), it does suggest that a common pooling process was used to detect the two different targets.

### (e) Summation region

For simplicity, contrast was summed over the entire stimulus region in the modelling in figures 3*b* and 4*b*, but the question arises, what is the smallest region over which summation is required? Figure 6 shows the results of rerunning the model on the full-on-full stimulus and the ‘white’ checks-on-full stimulus for a pedestal contrast of 32% (30 dB), and varying the diameter of a circular (hard-edged) summation aperture at their centres. The figure plots the ratio of target increment thresholds for these two stimuli (i.e. summation). The main model (equation (3.1), filled diamonds) must sum over at least seven carrier cycles (vertical solid line; a diameter of two checks) before it reaches the level of summation shown by the six observers in figure 3 (horizontal solid line).

### (f) Minkowski pooling

A pragmatic framework that has been widely used in models of suprathreshold tasks is Minkowski pooling over the response differences of multiple mechanisms (Wilson & Gelb 1984). The response of each mechanism has the typical form: resp_*i*_=mech_*i*_ (stim)^2.4^/(*z*+mech_*i*_(stim)^2^). But when the analysis is restricted to conditions well above detection threshold, this reduces to: resp_*i*_=mech_*i*_(stim)^0.4^. In this case Minkowski pooling of response differences is given by

(3.2) $$\begin{array}{c} \text{Minkowski}\_\text{sum}(\text{ped},\;\text{test})=\\ \sum_{i=1:2n}{\left({\text{mech}}_{i}{\left(\text{ped}+\text{test}\right)}^{0.4}-{\text{mech}}_{i}{\left(\text{ped}\right)}^{0.4}\right]}^{m}{)}^{1/m}. \end{array}$$

Equation (3.2) was solved numerically for target contrast where Minkowski_sum( )=1, following retinal inhomogeneity and spatial filtering as before. With *m*=1, this model behaved in a very similar way to the main model (equation (3.1)) for the stimulus pair here (compare open and filled diamonds in figure 6). With *m*=2 (open triangles), the model reached human summation when pooling extended over 8 carrier cycles, but with *m*=4 (filled triangles) the model could not reach this level at any spatial extent. Further simulations (not shown) confirmed that this conclusion was not critically dependent on the compressive response exponent of 0.4. Thus, a fourth-root summation rule on response differences will not do for the contrast discrimination experiments here.^1^

### (g) Signal combination or PS?

A Minkowski exponent (*m*) of 4 is often justified by appealing to its close approximation to PS at detection threshold (Meese & Williams 2000). However, the fourth-root rule does not have general theoretical support (Tyler & Chen 2000) and if area summation by PS is to be addressed, more detailed treatment is needed. Tyler & Chen showed that when the number of excited mechanisms doubles to fill the attention window (the array of mechanisms monitored by the observer), then PS produces high levels of summation, close to *m*=2. To provide a direct test of whether PS could account for the summation found with our stimuli (full-on-full versus checks-on-full) under these ‘high summation’ conditions, we performed Monte Carlo simulations (appendix C). With a pedestal contrast of 0%, these showed that 5 dB of summation is attainable for our stimuli using a MAX operator, a linear transducer and an attention window that matches the full stimulus. However, this model also predicts a shallow psychometric function (Weibull *β*∼1.3), whereas the geometric means of $\hat{\beta }$ for the seven observers in figure 4*b* were $\hat{\beta }=3.53$ (*N*=52) and $\hat{\beta }=3.71$ (*N*=28), for the check and full stimuli, respectively. The slope of the model psychometric function can be made steeper by increasing uncertainty (the number of mechanisms contributing to the MAX operation), but this moves PS away from the high summation region (Tyler & Chen 2000). Another method is to introduce an accelerating contrast transducer. Using *C*^2.4^ (the contrast transducer of our model), the model psychometric slopes increased to *β*∼4 and *β*∼3 for the check and full stimuli, respectively. However, the level of summation dropped to 3.48 dB; significantly less than the 5.06 dB found in the experiment (*T*=4.69; *p*=0.003, d.f.=6; two-tailed).

## 4. General discussion

A long-standing view of spatial vision is that (i) spatial summation of luminance contrast in the central visual field is due to PS among independent mechanisms and (ii) this summation process is disabled above threshold. Both parts of this view are challenged by the work here. In a preliminary experiment we demonstrated that bowed spatial summation curves are consistent with a signal combination strategy over many grating cycles. Experiment 2 showed that spatial summation of contrast occurs both at and above detection threshold over at least seven carrier cycles. Experiment 3 supported the idea that our different targets tapped a common pooling process. Finally, we rejected a fourth-root rule (figure 6) and PS model of area summation (appendix C), leaving signal combination as the most likely candidate.

### (a) Alternative model formulations

The combination of a contrast transducer of $C_{i}^{p}$and area summation of signal and noise (appendix A) predicts the same area summation at threshold as linear summation following a contrast transducer of $C_{i}^{2p}$and late additive noise (e.g. see Foley *et al*. 2007). This is also the same as Minkowski summation over a contrast transducer $C_{i}^{p^{\prime }}$and a Minkowski exponent *m*=2*p*/*p*′, see §1*a*. Thus, when combined with the spatial filtering and retinal inhomogeneity outlined in appendix A, all three of these formulations produce the spatial summation shown by the thick solid curve in figure 2 (where 2*p*=4.8). However, these formulations do not generally make the same prediction for the slope of the psychometric function (*β*). From signal detection theory the slope of the *d*′ psychometric function is equal to the overall contrast response exponent, *ϕ*. Conversion to Weibull units gives *β*=1.3×*ϕ* (Tyler & Chen 2000), where *ϕ*=*p*, 2*p* or *p*′ in the three formulations above. The average value of the psychometric slope for the full stimuli in the three experiments here was $\hat{\beta }=3.6$, which is close to *β*=3.1 predicted by the first formulation. It is very different from *β*=6.2 predicted by the second formulation, suggesting that this arrangement is unlikely. The third formulation is usually used with a linear transducer giving: *p*′=1, *m*=4.8 and *β*=1.3. In this case *β* is far too low. Thus, a Minkowski formulation with a linear transducer is inadequate. By setting *β*=3.1 to match that predicted by our preferred formulation, we find a transducer *p*′=2.4 and Minkowski exponent *m*=2. This formulation slightly underestimates summation at threshold (thin curve in figure 2), but is plausible above threshold (figure 6) and might be a useful alternative to the main model here. However, it would need to be developed to include lateral interactions to handle the relation between the SS, SL and LL configurations of Meese (2004) and the checks-on-checks versus full-on-full comparison here (figure 3*a*).

### (b) Summation mechanisms and lateral suppression

Human performance was very well described here using equation (3.1). But how might this equation be expressed in the human brain? One possibility is that the elements of a spatial array of first-order mechanisms with contrast responses ∝*C*^2.4^ are summed by a higher-order contrast integrator that is suppressed by an overlapping spatial array of mechanisms with contrast responses ∝*C*^2.0^. Another possibility is that each element in the spatial array has contrast response ∝*C*^2.4^ and is inhibited by a signal pooled across the spatial array of mechanisms having responses ∝*C*^2.0^. This would produce first-order mechanisms with self-inhibition (Foley 1994), lateral inhibition (Snowden & Hammett 1998) and sigmoidal contrast responses (Legge & Foley 1980). Summing across the array would produce a higher-order contrast integrator consistent with equation (3.1). Furthermore, placing the limiting source of additive noise after the inhibition but before the final summation stage is consistent with our suggestion that noise is summed over area (appendix A), but that it can be treated as late (i.e. it is not suppressed along with the signal) in experiment 2 (see appendix B).

There is evidence for both types of convergence described above. Numerous studies have found suppression from the ends, flanks and entire surrounds of the first-order filters using psychophysical (Ejima & Takahashi 1985; Cannon & Fullenkamp 1991; Xing & Heeger 2000; Chen & Tyler 2001; Meese 2004; Petrov *et al*. 2005) and neurophysiological methods (Gilbert & Wiesel 1985; Born & Tootell 1991; DeAngelis *et al*. 1994). There is also single-cell evidence for spatial pooling over large retinal fields. Gilbert & Wiesel (1985) and DeAngelis *et al*. (1994) reported extensive spatial integration of contrast across bar length in layer 6 of V1 and von der Heydt *et al*. (1992) described a specialized class of cells in V1 and V2 that respond to periodic stimuli with several cycles. Pollen *et al*. (2002) found spatial summation up to 16 cycles of length and width in V4 for sine-wave gratings, though the form of summation (e.g. linear, quadratic, MAX rule) probably varies among cells (Gustavsen *et al*. 2004). Other work has found spatial mechanisms with large receptive fields that pool over more complex patterns, such as hyperbolic, radiating and concentric grating patterns (Gallant *et al*. 1993; David *et al*. 2006). And psychophysical work has found evidence for mechanisms that sum structural (Field *et al*. 1993; Wilson & Wilkinson 1998; Dakin 2001; Parkes *et al*. 2001; Meese & Holmes 2004; Motoyoshi & Nashida 2004; Kuai & Yu 2006) and motion information (Morrone *et al*. 1995) over large areas of the retina.

However, one problem remains with the scheme above, which supposes lateral suppression across the full range of target contrasts. Experiments using annular masks have found little (Petrov *et al*. 2005) or no (Snowden & Hammett 1998) lateral suppression in the fovea at detection threshold, yet suprathreshold influences from contrast in the surround are found in matching (Cannon & Fullenkamp 1991) and discrimination experiments (Foley 1994; Chen & Tyler 2001; Meese 2004; Tolhurst 2007). Our experiments here do not address this issue, but the answer might be that sophisticated psychophysical observers use different mechanisms in the various tasks and conditions that pertain to tap the same processes. Another possibility is that lateral suppression might arise after the limiting noise, in which case perceived contrast would be affected but not contrast detection thresholds (Solomon & Morgan 2006). However, this would not explain the effects of surround contrast on contrast discrimination (Foley 1994; Meese 2004). A further possibility is that lateral suppression might be implemented by modulation of self-suppression by the surround (Foley 1994; Meese *et al*. 2007). As self-suppression is negligible when the pedestal contrast is 0, we should not expect an annular mask to raise detection thresholds for a central target on this model. Further experiments are needed to clarify these issues.

Other details of the process here remain to be elucidated. For example, future work is needed to determine whether there are similar mechanisms selective for more complex patterns (Gallant *et al*. 1993; Wilson & Wilkinson 1998; Dakin & Bex 2001; Motoyoshi & Nashida 2004; David *et al*. 2006; Tyler & Chen 2006). Alternatively, the pooling here might be an instantiation of a more flexible process capable of responding to a wide range of stimuli (e.g. Field *et al*. 1993; Meese & Georgeson 2005), perhaps matching to particular objects, features or other image characteristics. In particular, work is needed to understand what controls the spatial extent of summation, which can be restricted to a single central disc (Meese 2004), but not the check regions here (figure 3*c*). In any case, it is now clear that spatial pooling of luminance contrast is much more pervasive than once thought, and that its behaviour is predicted by extending the footprint of a contrast gain control equation over several hypercolumns.

## Figures and Tables

**Figure 1:**
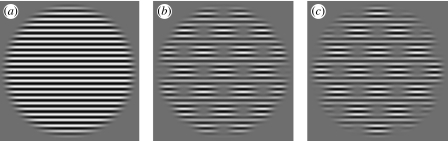
Stimuli used in experiments 2 and 3: (*a*) full stimulus (*b*) ‘white’ checks (*c*) ‘black’ checks. All three stimulus types served as pedestal (mask) and target in various combinations. They had a diameter of 9° displayed on a uniform square grey region with a width of 20.5° in the centre of the monitor. Closely related stimuli were used in experiment 1.

**Figure 2:**
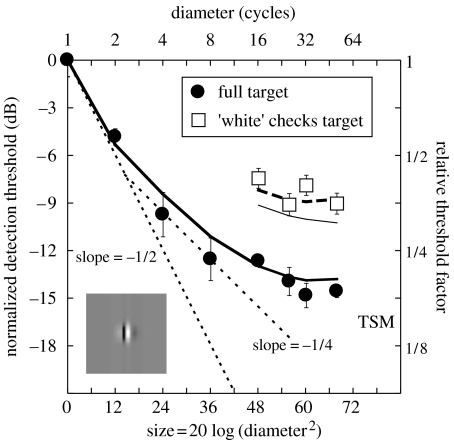
Area summation results from experiment 1 and model predictions (thick curves). Error bars show ±1 s.e. The inset shows the weighting function of the filter used in the modelling. The abscissa refers to the area bounded by the outer edge of the stimulus plateau. The dotted lines are fiducial contours with slopes of −1/2 and −1/4. The thin curve beneath the open squares is described in the Discussion.

**Figure 3:**
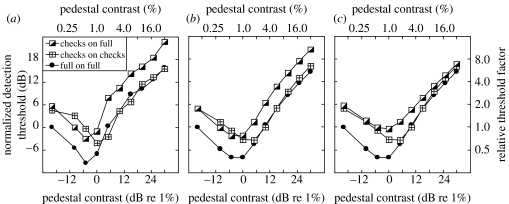
Contrast-masking (dipper) functions. (*a*) Results from experiment 2 averaged across two observers (L. M. and L. W.; approx. 800 or 1600 trials per point). The average standard error was 1.28 dB for L. M. and 0.92 dB for L. W. (*b*) Behaviour of the main model (using equation (3.1)). Two parameters (*z* and *k*; see appendix B) control the ‘dip’ and were adjusted to match the data by eye. (*c*) Matched model, the same model as in (*b*) except that the region of summation on the numerator of equation (3.1) is restricted to the high-contrast parts of the target in the checks-on-full condition. Data and models are normalized by the sensitivity to the full stimulus with 0% pedestal contrast (left-most points).

**Figure 4:**
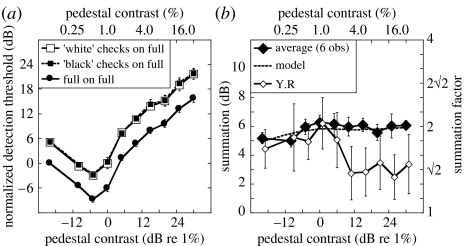
Results from experiment 2 where the pedestal was always a full stimulus. Error bars show ±1 s.e. across observers, or for Y.R as appropriate. (*a*) Dipper functions averaged across six observers. (*b*) Sensitivity ratios for full target and average of ‘black’ and ‘white’ checks for the six observers in (*a*) (approx. 7200 trials per point) and a seventh observer Y.R. The function for Y.R is offset laterally by 1 dB for clarity. The model prediction (using equation (3.1)) is shown by the dashed curve.

**Figure 5:**
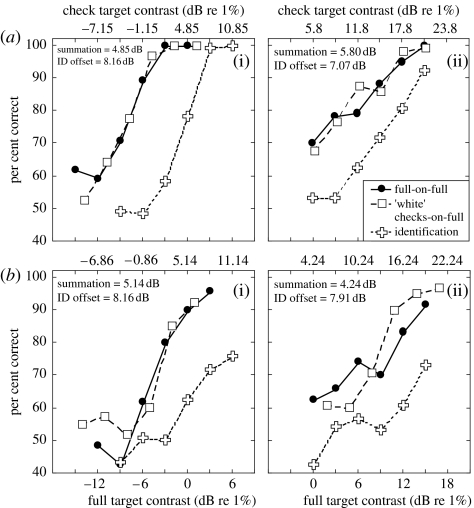
Detection and identification of ‘white’ checks and full targets for (*a*(i)(ii)) T.S.M. and (*b*(i)(ii)) R.J.S. for pedestal contrasts of (*a*(i),*b*(i)) 0% and (*a*(ii),*b*(ii)) 20%. The insets report spatial summation (the difference between the upper and lower contrast axes dB) and the lateral shift of the identification threshold relative to the detection threshold of the full stimulus (ID offset). The average slopes of the psychometric functions at detection threshold were $\hat{\beta }=3.2$ and $\hat{\beta }=3.08$ for detection and identification, respectively. For the 20% pedestal, they were $\hat{\beta }=1.5$ and $\hat{\beta }=1.9$.

**Figure 6:**
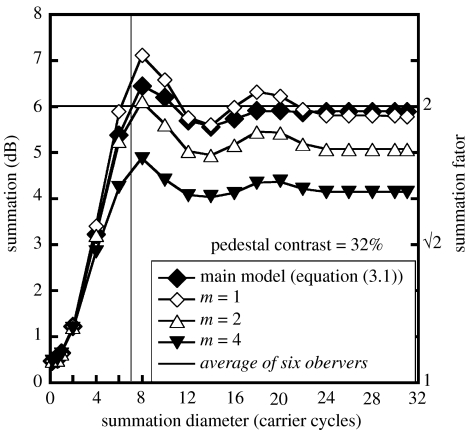
Summation levels as functions of the diameter of a circular summation aperture. Different symbols are for different models (see text for details).

**Table 1:** Predicted summation ratios (SRs) and slopes of the psychometric function for two different forms of summation (MAX and linear sum) with linear and accelerating contrast transducers at threshold and a compressive transducer above threshold.

Model	pedestal contrast (%)	SR (dB)	Weibull *β* checks	Weibull *β* full
MAX, linear	0	5.0	1.4	1.2
MAX, *C*^2.4^	0	3.5	4.0	3.0
MAX, *C*^0.4^	32	5.2	1.3	1.1
Σ, linear	0	6.0	1.3	1.3
Σ, *C*^2.4^	0	4.8	3.1	3.1
Σ, *C*^0.4^	32	5.9	1.3	1.3

## Endnote

1 The generally high levels of summation found in the models are due largely to the smooth modulation in the checks stimuli. The ‘black’ and ‘white’ checks physically sum to produce the full stimulus, but there is spatial overlap between them, which means that the mechanisms they stimulate also overlap. This enhances Minkowski summation beyond that found with independent mechanisms.

## Appendix A Model for experiment 1

Images were sampled with a resolution of 10 pixels per carrier cycle and multiplied by an attenuation surface to simulate the effects of retinal inhomogeneity. This surface was derived from the experiments of Pointer & Hess (1989). It is the product of a sensitivity loss of 0.3 dB per carrier cycle in the horizontal meridian (*x*-coordinate) and 0.5 dB per cycle in the vertical meridian (*y*-coordinate). The attenuated image was filtered by a pair of quadrature log-Gabor filters (Meese & Georgeson 2005) with spatial frequency bandwidth of 1.6 octaves and orientation bandwidth of ±25°, which are typical in the literature (DeValois & DeValois 1990). The filters were matched to the spatial frequency and orientation of the carrier grating, and their outputs were full-wave rectified and scaled to the range 0–1. Linear summation was performed across the quadrature filters after nonlinear transduction (Legge & Foley 1980) and across the stimulus region defined by the half-height of its envelope. (We assume that the observer could identify the target region on each trial, consistent with the use of a blocked design.) Unit-variance, Gaussian noise was added to each of the *n* mechanisms (pixels) in this region (where *n* is proportional to the square of the target's diameter). Thus, the SNR for the summation process is given by

(A1) $$\text{SNR}=\frac{\sum_{i=1:n}\left(|C_{\text{stim}}\times {\text{sfilt}}_{i}{|}^{2.4}+|C_{\text{stim}}\times {\text{cfilt}}_{i}{|}^{2.4}\right)}{\sqrt{2n}},$$

where *C*_stim_ is the Michelson contrast of the carrier grating (in the range 0–1) and sfilt_*i*_ and cfilt_*i*_ are the contrast responses of the quadrature filters to unit contrast at the *i*th location in the image. Assuming a criterion SNR of unity at detection threshold, equation (A1) rearranges to

(A2) $$C_{\text{thresh}}={\left[\frac{\sqrt{2n}}{\sum_{i=1:n}\left(|{\text{sfilt}}_{i}{|}^{2.4}+|{\text{cfilt}}_{i}{|}^{2.4}\right)}\right]}^{1/2.4}.$$

See appendix SA in electronic supplementary material 1 for further details.

## Appendix B Model for experiment 2

The model from appendix A was extended to operate the above detection threshold as follows:

(B1) $$\text{resp}(\text{stim})=\frac{\sum_{i=1:n}\left(|\text{sfilt}C_{i}{|}^{2.4}+|\text{cfilt}C_{i}{|}^{2.4}\right)}{z+\sum_{i=1:n}\left(|\text{sfilt}C_{i}{|}^{2}+|\text{cfilt}C_{i}{|}^{2}\right)},$$

where sfilt*C*_*i*_ and cfilt*C*_*i*_ are the contrast responses of the quadrature filters to the pedestal plus target stimuli as appropriate. The decision variable was given by SNR=*k*=resp(ped+test)−resp(ped) at detection threshold for the target, where *ped* and *test* are the pedestal and target stimuli, and *k* is a sensitivity parameter.

In experiment 2, where the stimuli had equal diameters, the spatial extent of model summation was the same across conditions. Therefore, the noise level was constant across conditions and was absolved by *k*. The model equations were solved numerically for target contrast over a range of pedestal contrasts to derive masking functions for each stimulus.

The filtering (to give sfilt*C* and cfilt*C*) followed multiplication of the stimulus with the attenuation surface, as before, though this was not critical. See appendix SB in electronic supplementary material 1 for further details.

## Appendix C PS for experiment 2

Monte Carlo simulations were used with stochastic noise to make PS predictions for stimuli used in experiment 2. As we are interested in the distribution of contrast (responses) over space, the stimulus envelope (*env*) was treated as the signal over an area equal to two neighbouring checks. In this scheme, Michelson contrast corresponded with the peak of the distribution for the check stimuli, and the height of an entirely uniform distribution for the full stimuli. Zero-mean, unit-variance, Gaussian noise (*G*) was added to each mechanism independently on each 2IFC interval of each simulated trial after contrast transduction (see below) to give resp_*i*_+*G*_*i*_, for the *i*th mechanism in the array. The observer was assumed to monitor the contents of the array equivalent to two checks without repetition (1953 mechanisms, though this is not critical) on both intervals of every trial. The response to each interval was given by the maximum response in the array (MAX[resp_*i*_+*G*_*i*_]), and on each trial the simulated observer selected the interval with the maximum response (Tyler & Chen 2000). This was done for a wide range of target contrasts (*C*_test_) placed in 0.5 dB steps, with 2000 simulated trials at each level. Weibull functions (Quick 1974) were fitted to the simulated data to calculate threshold (the contrast at 75% correct) and the slope of the psychometric function (the Weibull parameter *β*). This was done for a check stimulus and a full stimulus to predict a summation ratio (SR). The simulations were also done using linear summation of responses $\left(\sum_{i=1:n}\left[{\text{resp}}_{i}+G_{i}\right]\right)$ instead of the MAX operator.

The simulations were run with a pedestal contrast (*C*_ped_) of either 0 or 32%, where the pedestal was always a full stimulus. They were also done for three different contrast transducers. A linear transducer (where pedestal contrast is immaterial), an accelerating transducer (resp_*i*_=[env_*i*_*C*_test_]^2.4^; for a pedestal contrast of 0%) and a compressive transducer (resp_*i*_=[env_*i*_(*C*_test_*+C*_ped_)]^0.4^) for a pedestal contrast of 32%. Predicted SRs and slopes of the psychometric function (*β*) are shown in table 1. See appendix SC in electronic supplementary material 1 for further details.

## Supplementary Material

Extended appendices Appendices SA, SB and SC, enhanced versions of the appendices A, B and C in the main document

Data for individual observers Individual results for the seven observers in Experiment 2

Stimuli for experiment 1 The stimuli that were used in Experiment 1
