# Supplementary material for: Area summation in human vision at and above detection threshold
Source: Proc Biol Sci. 2007 Sep 13;274(1627):2891–900. doi: 10.1098/rspb.2007.0957 (PMC2211515; doi:10.1098/rspb.2007.0957)

Supplement 2 of 3: Individual results for the seven observers in Experiment 2

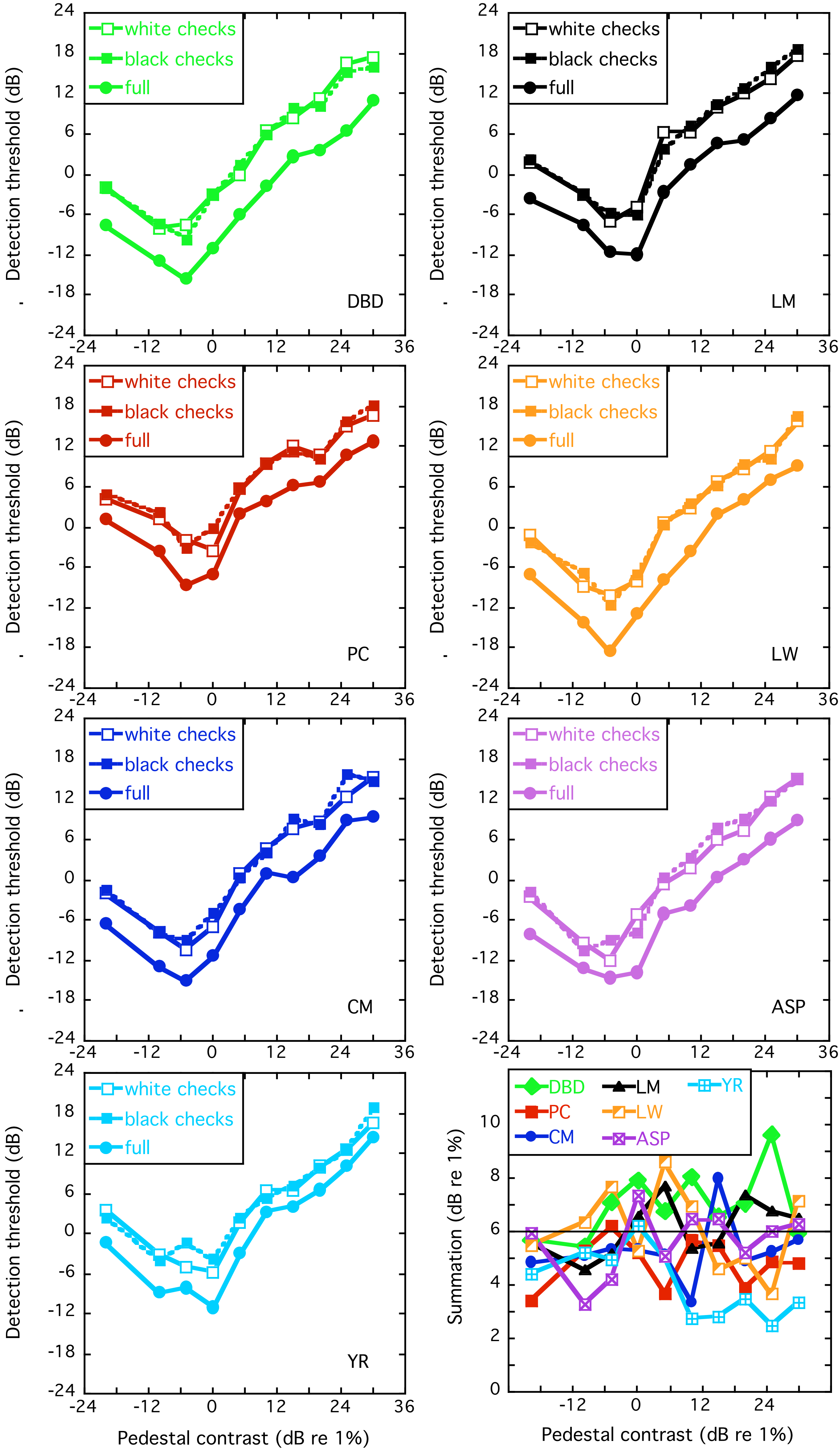

Supplement: Supplementary file 1 [file rspb20070957s21.pdf]
