# Supplementary material for: Area summation in human vision at and above detection threshold
Source: Proc Biol Sci. 2007 Sep 13;274(1627):2891–900. doi: 10.1098/rspb.2007.0957 (PMC2211515; doi:10.1098/rspb.2007.0957)
Supplement: Supplementary file 2 [file rspb20070957s20.rtf]

Supplementary material for Meese & Summers (2007), Proceedings of the Royal Society B.

Supplement 1 of 3.

In this supplement we provide enhanced versions of the appendices (A, B C) in the main document. 

Appendix SA: Model for Experiment 1
Here we develop the model used in Experiment 1 (detection thresholds), and illustrate the step-by-step effects of its various components. Images were sampled with a resolution of 10 pixels per carrier cycle (though this was not critical) and multiplied by the attenuation surface shown in Fig A1d, to simulate the effects of retinal inhomogeneity. This surface was derived from the empirical results of Pointer and Hess (1989). It is the product of a sensitivity loss of 0.3dB per carrier cycle in the horizontal meridian (x co-ordinate) and 0.5 dB per cycle in the vertical meridian (y co-ordinate). The attenuated image was then filtered by a pair of quadrature The use of two filters in a quadrature pair was not critical. Very similar results were found using either filter phase alone. Furthermore, the order in which retinal inhomogeneity and spatial filtering are applied has negligible impact on the model's behaviour.  log-Gabor filters (Meese & Georgeson, 2005) with spatial frequency bandwidth of 1.6 octaves and orientation bandwidth of ±25° (bandwidths at half-height; see inset in Fig 2). These bandwidths are within normal bounds in the psychophysical and neurophysiological literature (De Valois & De Valois, 1990). The filters were matched to the spatial frequency and orientation of the carrier grating and their outputs were full-wave rectified and scaled to the range 0 to 1 (where unity was the maximum possible response). Each pixel value was then raised to a power of 2.4 (Legge & Foley, 1980) to represent the accelerating contrast-response nonlinearity of the mechanism at each pixel location. Linear summation was performed across the quadrature filters and across the stimulus region defined by the half-height of its envelope (i.e. a diameter of pixels; where cycles is the number of carrier cycles in the plateau region of the full stimulus). Unit-variance, Gaussian noise was added to each mechanism (pixel), giving a deterministic noise level of √(2n) for each image, where n is the number of mechanisms (pixels) in the luminance modulated part of the image (i.e. , where r is the radius of the target). Thus, the signal to noise ratio (SNR) for the summation process is given by:

,			(SA1)

where C_stim_ is the Michelson contrast of the carrier grating (in the range 0 to 1), and sfilt_i_ and cfilt_i_ are the sine- and cosine-phase filter responses at the ith of n pixels in the image, scaled to a response range of –1 to 1. Assuming a criterion signal to noise ratio (SNR) of unity at detection threshold, it follows that:

, 					(SA2)

which rearranges to give:

.				(SA3)


As filter gain parameters have been omitted for simplicity, the contrast units of C_thresh_ are arbitrary. These arbitrary units cancel when the model predictions are normalized to the response to the smallest stimulus.
Fig SA1 shows the response of the model to 'full' stimuli through the various stages of development (see figure caption for details). 


Fig SA1. Development of the detection model. (A) The pale line shows ideal summation of signal and noise for a linear transducer (equivalent to quadratic summation of signal). It has a slope of –1/2 on double log co-ordinates. The red curve shows the affect of spatial filtering. This increases the initial part of the summation slope but has little effect on the rest of the function. This happens because of the blurring effect of the filter's weighting function, which has its greatest impact on the effective size of the smallest stimulus. (B) The effect of adding a nonlinear contrast transducer (, where p = 2.4). (C) The effect of adding retinal inhomogeneity. This was done by multiplying the stimulus by the attenuation surface in (D) before filtering. The curve in (C) is the one plotted in Fig 2 (of the main document) for the full stimulus. The same model was used for the dashed curve in Fig 2, but the stimuli were 'white' checks. 

Fig SA2 is a replot of Fig 2 from the main body of the paper. It shows data from Experiment 1 and the model predictions using Eq SA3 (thick curves). An identical prediction is made for the full stimulus (solid thick curve) by a model in which the transducer is linear, the early noise is omitted (i.e. the same late additive noise is placed on the output for all stimulus sizes), retinal inhomogeneity remains intact and a Minkowski exponent of m=4.8 is used. (4.8 is the product of p = 2.4 and a further exponent of 2 equivalent to ideal summation of signal and noise). Thus, Eq SA1 is replaced by:

,				(SA4)

giving:

 	.						(SA5)

The prediction of this model for the check stimulus is shown in Fig SA2 by the thick dotted curve just below the open square symbols. Summation is only slightly less than that found in the experiment. However, this model predicts a psychometric function that is much shallower (Weibull β~1.3) than those found in typical detection experiments (β~3 or 4). The average slope of the psychometric function for the full stimulus in Fig SA2 was β = 3.8, a little steeper than the prediction of β = 3.1 made by the main model in Table SC1.


Fig SA2. The same as Fig 2 in the main body but with a model prediction (Eq SA5) for a linear transducer and Minkowski exponent m = 4.8 (thick solid curve and dotted curve).


Appendix SB: Main model for Experiment 2
The model from Appendix A was extended to operate above threshold (Meese, 2004) by including suppressive contrast gain control (Heeger, 1992; Foley, 1994) as follows:

, 					(SB1)

where sfiltC_i_ and cfiltC_i_ are the contrast-responses of the quadrature filters to the pedestal plus test stimuli as appropriate (scaled to the range –1 to 1 for the minimum and maximum responses).
Further:

 					(SB2)

at detection threshold for the test where ped and test are the pedestal and test stimuli and k is a sensitivity parameter. 
In Experiment 2, where the stimuli had equal diameters, the spatial extent of model summation was the same across conditions. Therefore, the noise level was constant across conditions and was absolved by the criterion response parameter k in Equation SB2. Equations SB1 and SB2 cannot be rearranged to form an analytic expression for the dependent variable and were solved numerically for test contrast over a range of mask contrasts to derive masking functions for each stimulus. These were normalized to a detection threshold of 0dB for the condition where mask contrast was 0% for the full stimulus. The parameters z and k were adjusted by hand to provide an acceptable match to the data. These control the 'dip' and were the only free parameters in the model.  
Note that the filtering (to give sfiltC and cfiltC) followed multiplication of the stimulus with the attenuation surface (Fig SA1d), as before, though this was not critical. 


Appendix SC: Probability summation for Experiment 2
Monte Carlo simulations were used with stochastic noise to make probability summation (PS) predictions for the stimuli in Experiment 2. As we are interested in the distribution of contrast (responses) over space, the stimulus envelope (env) was treated as the signal over an area equal to two neighbouring checks. Each check was sampled by a 32x32 array (to the boundaries of the check). In this scheme, Michelson contrast corresponded with the peak of the distribution for the check stimuli, and the height of an entirely uniform distribution for the full stimuli. Zero-mean, unit-variance, Gaussian noise (G) was added to each mechanism independently on each 2IFC interval of each simulated trial after contrast transduction (see below) to give resp_i_+G, for the ith mechanism in the array. The observer was assumed to monitor the contents of the array equivalent to two checks without repetition (32-1)x(2x32-1) on both intervals of every trial. That's 1,953 mechanisms, though the precise number is not critical. Increasing the number of checks in multiples of two produces identical results. The response to each interval was given by the maximum response in the array (MAX[resp_i_+G]), and on each trial the simulated observer selected the interval with the maximum response (Tyler & Chen, 2000). This was done for a wide range of test contrasts (C_test_) placed in 0.5dB steps, with 2000 simulated trials at each level. Weibull functions (Quick, 1974) were fitted to the simulated data to calculate threshold (the contrast at 75% correct) and the slope of the psychometric function (the Weibull parameter β). This was done for a check stimulus and a full stimulus to predict a summation ratio. The simulations were also done using linear summation of responses () instead of the MAX operator, confirming several of the deterministic predictions from Appendix SB (see Table SC1). 
	The simulations were run with a pedestal contrast (C_ped_) of either 0% or 32%, where the mask was always a full stimulus. They were also done for three different contrast transducers. A linear transducer (where mask contrast is immaterial), an accelerating transducer (resp_i_=[env_i_C_test_]^2.4^) (for a mask contrast of 0%), and a compressive transducer (resp_i_=[env_i_(C_test_+C_ped_)]^0.4^) for a mask contrast of 32% (the highest mask contrast in the experiments). Predicted summation ratios (SR) and slopes of the psychometric function (β) are shown in Table SC1. 

Model	Mask
contrast 	SR (dB)	Weibull
β 
Plaid checks	Weibull
β 
Full	
MAX, linear	0%	5.00	1.4	1.2	
MAX, C^2.4^	0%	3.48	4.0	3.0	
MAX, C^0.4^	32%	5.23	1.3	1.1	
Σ, linear	0%	6.05	1.3	1.3	
Σ, C^2.4^	0%	4.79	3.1	3.1	
Σ, C^0.4^	32%	5.92	1.3	1.3	
Table SC1. Predicted summation ratios (SR) and slopes of the psychometric function for two different forms of summation (MAX and linear sum) with linear and accelerating contrast transducers at threshold, and a compressive transducer above threshold. A bootstrapping technique was used to calculate 95% confidence intervals of the estimates. These were always < 0.21dB for the SR and < 0.14 for β.

As some readers might find some of these results surprising (e.g. ~5dB of summation using a MAX rule), we validated the computations by performing the analysis for a more intuitive situation where the modulation was a square-wave (i.e. where env_i_ = 0 and 1, for the black and white checks respectively). This allows the calculations to be compared with values expected from previous work in several situations (see Table SC2 and caption for details). 
The generally high levels of model summation in Table SC1 are due largely to the smooth modulation in the checks stimuli. This enhances Minkowski summation and other forms of nonlinear summation beyond that found with independent components and mechanisms. 

Model	Mask
contrast	SR (dB)
Calculated	SR
(dB)
Expected	Weibull
β 
Square checks	Weibull
β 
Full 	
MAX, linear	0%	3.01	2.86	1.5	1.2	
MAX, C^2.4^	0%	1.25	?	3.4	3.1	
MAX, C^0.4^	30%	3.14	~2.86	1.3	1.2	
Σ, linear	0%	6.06	6.02	1.3	1.3	
Σ, C^2.4^	0%	2.54	2.51	2.9	3.1	
Σ, C^0.4^	30%	6.27	~6.02	1.3	1.3	
Table SC2. As Table SC1, but for a square-wave modulator instead of a plaid. From top to bottom, the expected values were derived as follows: (i) Table 1 in Tyler and Chen (2000). (ii) Previously unknown. (iii) The situation approximates linearity for small signals and is expected to be similar to the MAX, linear entry. (iv) The linear sum of two equal signals in constant noise increases the signal by a factor of 2 (6dB). (v) Equivalent to Minkowski summation with an exponent of 2.4. (vi) The situation approximates linearity for small signals and is expected to be similar to the Σ, linear entry.
